# The Future of Protein Secondary Structure Prediction Was Invented by Oleg Ptitsyn

**DOI:** 10.3390/biom10060910

**Published:** 2020-06-16

**Authors:** Daniel Rademaker, Jarek van Dijk, Willem Titulaer, Joanna Lange, Gert Vriend, Li Xue

**Affiliations:** 1 Centre for Molecular and Biomolecular Informatics (CMBI), Radboudumc, 6525 GA Nijmegen, The Netherlands; daniel.rademaker@radboudumc.nl (D.R.); jarek.vd@gmail.com (J.v.D.); willem.168@hotmail.com (W.T.); vriendgert@gmail.com (G.V.); 2 Bio-Prodict, 6511 AA Nijmegen, The Netherlands; joasialange@gmail.com; 3 Baco Institute of Protein Science (BIPS), Mindoro 5201, Philippines

**Keywords:** Oleg Ptitsyn, secondary structure prediction

## Abstract

When Oleg Ptitsyn and his group published the first secondary structure prediction for a protein sequence, they started a research field that is still active today. Oleg Ptitsyn combined fundamental rules of physics with human understanding of protein structures. Most followers in this field, however, use machine learning methods and aim at the highest (average) percentage correctly predicted residues in a set of proteins that were not used to train the prediction method. We show that one single method is unlikely to predict the secondary structure of all protein sequences, with the exception, perhaps, of future deep learning methods based on very large neural networks, and we suggest that some concepts pioneered by Oleg Ptitsyn and his group in the 70s of the previous century likely are today’s best way forward in the protein secondary structure prediction field.

## 1. Introduction

In 1969, Oleg Ptitsyn published the article “Statistical analysis of the distribution of amino acid residues among helical and non-helical regions in globular proteins” [1]. This article forms the bridge between visual inspection of the (few) available protein structures to learn about their fold, stability, and (local) structure characteristics, and the protein secondary structure prediction (PSSP) methods of the 70s. The oldest article about PSSP we could find in PubMed is by Ptitsyn et al. [2] in 1973, albeit older articles from his group were already published in Russian in 1970 [3,4]. 

Thousands of articles have been written about PSSP. In the beginning, these studies were mainly performed to deepen our understanding of the principles of protein folding and stability. More recently, however, PSSP became mostly a topic to teach bioinformatics master students how to write software, or to illustrate that a newly developed machine learning technique can be made useful.

The predicted secondary structure of proteins seems to have very little practical use. Manual inspection of the first 500 hits obtained by a Google Scholar search for “protein secondary structure” revealed a couple dozen articles about either the determination of secondary structure by—or the use of the known secondary structure in cryo-EM, atomic force microscopy, CD, IR, NMR, ESR, and Raman and vibration spectroscopy based biophysical studies. Additionally, we found “protein secondary structure” mentioned in articles related to spray-drying proteins [5], micelle density [6], protein design [7], aging of dried pollen [8], biofilm formation [9], dried seeds [10], and the effect of aspirin [11], as well as in several articles related to protein folding, protein–protein interfaces, and the stability of secondary structure elements. In bioinformatics studies, such as molecular dynamics simulations, fold recognition, structure superposition, or clustering protein structures in other ways, secondary structure can be used to evaluate or visualize the results. Fold recognition is the only example we found in which predicted secondary structures are sometimes used as an essential aspect of the method. In the past, one of us (G.V.) used predicted secondary structure in a project aimed at designing epitopes for peptide-based antibody generation, and in a project aimed at designing thermosensitive mutations, but these studies were not published. The vast majority of the 500 manually inspected articles describe methods to predict the secondary structure of a protein given its sequence. The method designed by Chou and Fasman [12,13] in the early 70s, for example, was first implemented in computer code by Lenstra in 1977 [14]; the last implementation we found was published 36 years later [15].

Most articles that describe yet another PSSP program start with a sentence like: “The ability to predict local structural features of a protein from the sequence is of paramount importance for unravelling its function in absence of experimental structure information”, which simply is nonsense as the function of a protein cannot be determined from its 3D structure, and thus even less from its (predicted) secondary structure. 

We found a series of articles in which the authors determine the secondary structure of proteins from their three-dimensional coordinates, but it is clear that DSSP [16] is the de facto standard for this work, and in this article, we only use DSSP to assign the secondary structure from 3D coordinates. 

Yang et al. [17] recently reviewed the 65-year history of secondary structure predictions rather beautifully. They start with the work of Pauling, Corey, and Branson [18,19], and end with today’s deep-learning methods that seem to outperform everything done in the previous 64 years. We can only urge the reader to read that review before continuing reading this article. Yang et al. end with four conclusions: (1) The accuracy of state-of-the-art three-state methods is around 83%; (2) Recent improvements come from larger databases, the use of templates, and powerful deep learning techniques; (3) Prediction of backbone angles (which can help predicting 3D structures from sequence alone) has room for further improvement; (4) The future (of PSSP) is bright as next-generation deep learning techniques can remember long-range interactions.

**Figure 1:**
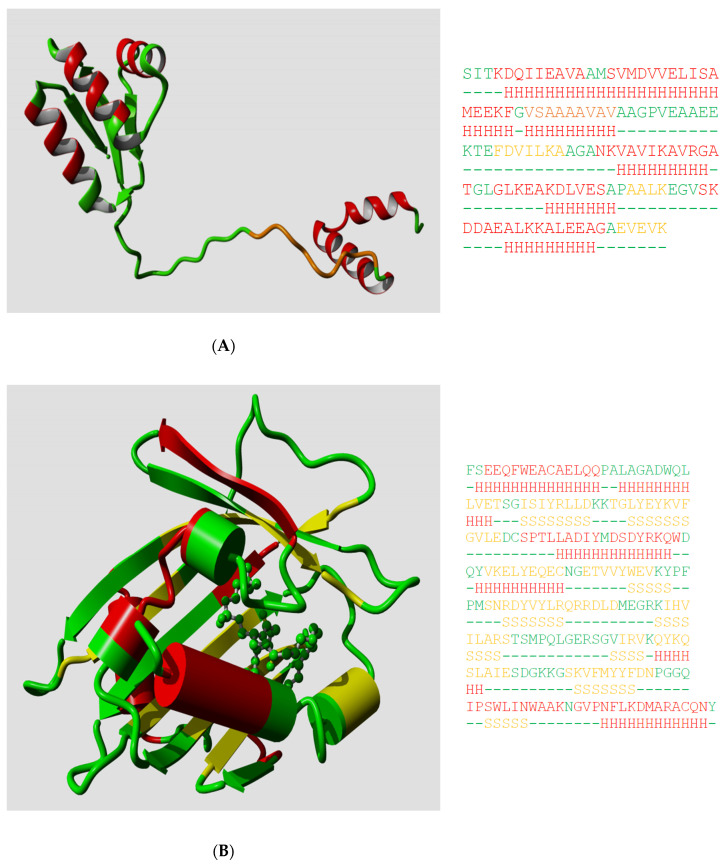
(**A**) The structure of one monomer of the L7/L12 protein dimer. PDBid 1rqu [20] is colored by the helix predictions made by Ptitsyn et al. in 1973 (helix = red; nonhelix = green). The small orange stretch was predicted as helix by Ptitsyn et al., but falls in a region for which no NMR data is available, but for which Bocharov et al. [20] wrote “There is an indirect evidence of the existence of transitory helical structures at least in the first part (residues 33–43) of the hinge region”. To the right the sequence is shown colored by its secondary structure with underneath it the predicted secondary structure. Helices are green, strands are yellow, and everything else is green. Loops are shown as dashes. (**B**) The structure of the phosphatidylcholine-transfer protein from bovine liver. PDBid 1ln3 [21] is colored by the secondary structure predictions by Akeroyd et al. [22] using the methods of Chou and Fasman, and Lim as implemented by Lenstra [14]. (same coloring as in A). Many things can be said about this prediction, but not that it could have been useful for any practical purpose. The sequence and predicted secondary structure to the right are also colored as in A.

Ptitsyn, Denesyuk, Finkelstein, and Lim based their landmark prediction of the secondary structure of the L7, L12 *Escherichia coli* ribosomal protein [2] on fundamental concepts of polymer physics for helix–coil transitions in water-soluble synthetic polypeptides. Today, the structure of the L7, L12 protein is known [20], so we can see how well they did in 1973. Ptitsyn et al. started from the assumption that the protein was mainly helical and contained no strands; this was based on biophysical measurements that, in retrospect, were wrong. Consequently, they missed the three strands in their prediction, but as Figure 1A shows, their helix predictions were remarkably accurate. One of us (G.V.) was involved (in 1982) in the prediction of the secondary structure of phosphatidylcholine-transfer protein from bovine liver using Lenstra’s implementation of two PSSP methods [14]. Figure 1B shows that this prediction at best can be called poor now that we can map it on the structure of a close homolog. 

The methods developed by Ptitsyn et al. were soon extended, refined, and published by Lim [23]. Unlike Ptitsyn and Finkelstein, who mainly paid attention to residue propensities to be inside or at the ends of secondary structures, Lim concentrated on hydrophobic and hydrophilic surfaces of these structures. It should be mentioned that both these rather different methods, Ptitsyn–Finkelstein’s and Lim’s, performed well in the first world-wide PSSP “competition” [24], which one could call “CASP-0”. Lim’s article is hard to read, and the method is even harder to implement in a computer program.

**Figure 2:**
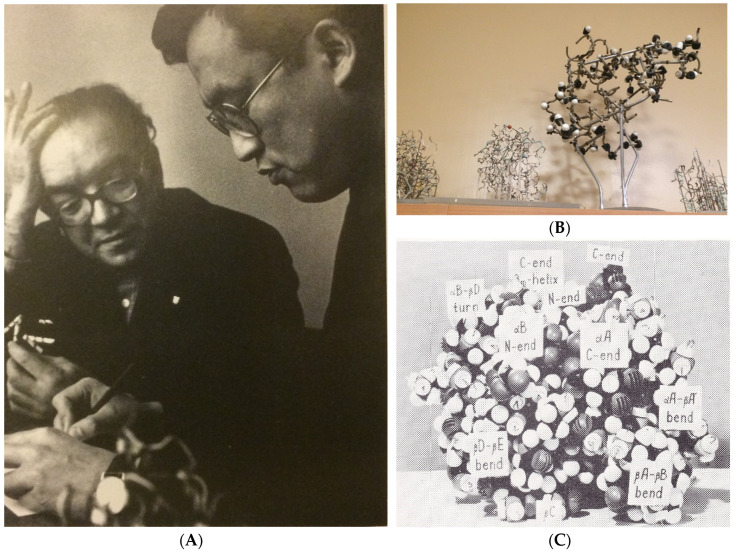
The early history of protein secondary structure prediction. (**A**) Oleg Ptitsyn (with hand in hair) and V.I. Lim study physical models of proteins and peptides. (**B**) Recent photo of some of the wire models used in Ptitsyn’s lab before (and also after) computer graphics systems became available. (**C**) Model of one of the early structure predictions by Ptitsyn’s group. One of us (G.V.) spent time in the early 90s with Ptitsyn and Finkelstein looking at these models, at which time Oleg told him that “looking at models is the best theoretical approach”, a lesson G.V. never forgot and taught all his students, including most coauthors on this article.

The concept of Jackknifing or at least using disjunct training and testing datasets was not yet widely known in those days and consequently Lim claimed that the method was 80–85% correct. Kabsch and Sander have later corrected this number to ~56% [25]. They described the lack of Jackknifing as “The difference can be understood to be due to special rules tailored to particular proteins in Lim’s method”. The people in Ptitsyn’s group actually spent time looking at wire-models and space-filling models of protein structures (see Figure 2). The small set of structures available to them in those days made that the rules they saw as “general” actually were often rather specific for particular (classes of) proteins. In retrospect, it is not clear whether Ptitsyn initially focused on the prediction of helices because the protein structures available around 1970 contained many more residues in helices than in strands, or because helices are easier to understand in terms of protein physics. More recent PSSP methods usually are aiming to predict the secondary structure of all proteins, and although examples probably exist, the rule is that people who publish a PSSP method have not looked at any structure they predicted.

From the hundreds of PSSP methods that have been published, the method by Chou and Fasman (C&F) [12,13] definitely became the most famous, together with the methods of Lim [23], and Garnier–Osguthorpe–Robson [26]. For many years, new methods were published with the main conclusion of how much (actually how little) better they worked than these three methods. Chou and Fasman simply counted how often each amino acid type were observed in each of the four secondary structure types (helix, strand, turn, loop), and thereby derived rules for PSSP. Like the method of Lim, the C&F method suffers from a problem that Kabsch and Sander described as “There are ambiguities in two of the best known methods, those of Chou and Lim, in that they often give different results in the hands of different people and are therefore not programmable without extension or modification”.

The first serious breakthrough in PSSP came when Rost et al. used neural networks with multiple sequence alignments as input [27]. We expect the second major breakthrough to come from deep neural networks (which essentially are, or according to us should be, very large neural networks (e.g., [28])).

The upper limit of predictability of secondary structure has often been discussed (e.g., [29]), and it is commonly accepted that this upper limit is around 90%. We will shed some extra light on this topic. We find hints in the literature (e.g., [29]), about the lack of generality of PSSPs, and we will address this topic with some detail, to arrive at the conclusion that the work of the real, biological neural network of Oleg Ptitsyn, simulated in today’s artificial neural networks, holds the best hope for the future of PSSPs.

## 2. Materials and Methods

DSSP was used for all secondary structure assignments. This software, and the assignments for all 3D protein structures in the PDB, are freely available from the CMBI protein structure bioinformatics facilities website [30]. The list of dimers in the PDB was determined with WHAT IF [31]. This list is available from https://swift.cmbi.umcn.nl/gv/facilities/too. PDB-REDO [32] files (freely available from https://pdb-redo.eu/) are reinterpreted PDB files that are re-refined with today’s software and today’s CPU power. It was shown convincingly that a PDB-REDO file normally is a better model for the underlying macromolecule than the corresponding PDB file [33].

To classify proteins based on the predictability of their secondary structures, we designed a secondary structure normality score (SSN score). Our SSN score is based on C&F-like parameters. C&F-like parameters for single amino acids and for amino acid pairs were determined with the proprietary software CFcheck that is available from the CMBI’s github area (https://github.com/cmbi/CF-check). This software determines the secondary structure preference parameters $P_{1}\left(AA_{i},\;SS_{k}\right)$ and $P_{2}\left(AA_{i},\;AA_{j},SS_{i},\;SS_{j}\right).\;P_{1}\left(AA_{i},\;SS_{i}\right)$ is the likelihood of amino acid type $AA_{i}$ appearing in secondary structure state $SS_{i}$. DSSP secondary structure states were reduced to three states (helix, strand, rest); and $P_{2}\left(AA_{i},\;AA_{j},SS_{i},\;SS_{j}\right)$ is the likelihood of $AA_{i}$ and its C-terminally adjacent neighbor $AA_{j}$ appearing in secondary structure states $SS_{i},\;SS_{j},\;\mathrm{respecitvely}.$ Box 1 explains how these parameters are determined, and how they are used. Although mathematically “cleaner” than the method published by Chou and Fasman [12,13], the use of P_1_ and P_2_ parameters gives similar results. The advantage of preference parameters over the original Chou and Fasman parameters is that preference parameters for individual amino acids and pairs can simply be added up over a whole protein. The secondary structure of a protein with a high SSN score will be predicted well by C&F like methods, and probably by most PSSP methods. A low SSN score implies poor predictability of the secondary structure.

We define the secondary structure normality (SSN-score) of a protein of n amino acids as the average of all n P_1_ and n−1 P_2_ values. SSN-scores were determined for 22,405 protein chains longer than 49 amino acids that share no pair-wise sequence identity larger than 30% [34]. An SSN score measures how “normal” a protein’s secondary structure is in terms of amino acid compositions in secondary structures. A protein has a high SSN score when the amino acid composition of its secondary structures is similar to the average of 22,405 proteins.

SSN-score frequency plots are scaled to have the same area under the curve. The distributions were fitted using the Parzen–Rosenblatt window method (https://en.wikipedia.org/wiki/Kernel_density_estimation#cite_note-Ros1956-1) as implemented in the Seaborn software (https://seaborn.pydata.org/generated/seaborn.kdeplot.html#seaborn.kdeplot).

For the analysis of intrinsically disordered proteins we have selected a subset of 225 sequences from the DisProt database [35] with a disorder content of at least 60%.

Box 1 **Preference parameter determination.**

60 Preference parameters are determined for the 20 amino acid types in 3 states and 3600 parameters are determined for 400 pairs of amino acid types in 3 × 3 states. These preference parameters are determined as the logarithm of the observed frequency of each (aa_i_,ss_j_) or (aa_i_,aan_i_,ss_j_,ssn_j_) combination divided by their predicted frequency. These predictions are the result of the multiplication of the chance that any amino acid type is aa_i_ with the chance that the secondary structure is ss_j_.

For example, with 7% of the amino acids in the training set being Leu and 35% of all amino acids being helical, the expected frequency F(Leu,H) is 7% of 35% which is about 2.5%. The number of leucines in helices is considerably higher than 2.5% of the total number of amino acids in the dataset and therefore F(Leu,H)obs/F(Leu,H)pred is larger than 1, and thus the preference parameter P_1_(Leu,helix) = log(F(Leu,H)obs/F(Leu,H)pred) is larger than 0.0. The calculation of P_2_ values go in a similar fashion.

**Preference parameter usage**. For each protein of length n, n P_1_ and n−1 P_2_ values are determined by simple lookup in the P_1_ and P_2_ tables. All so obtained preference parameter are added up and divided by (n+n−1) to arrive at the protein’s SSN score. If this SSN score is high, many amino acids have the most preferred secondary structure, which also implies that C&F-like PSSP methods should be able to predict their secondary structure well.

## 3. Results

Oleg Ptitsyn studied secondary structures by looking at 2D and 3D images of proteins rather than analyzing numbers that describe protein structures. We picked three topics that illustrate that his approach to protein secondary structure prediction was a good one.

- How accurate are the determinations of secondary structure from 3D structures?
- How important is the size of the training set?
- How important is the composition of the training set?

We address these topics because they illustrate how the methods designed by Oleg Ptitsyn and his group in the 70s were actually ahead of their time, and we will conclude that deep learning can merge the best of both worlds by automatically combining large datasets, big computers, and everything else we learned about PSSP methods with the idea that one cannot apply one PSSP method to all classes of proteins. These three topics also illustrate Oleg Ptitsyn’s idea that understanding the laws of physics that apply to the formation of secondary structure elements is very important.

### 3.1. How Accurate Are the Determinations of Secondary Structure from 3D Structures?

Computer programs, such as DSSP, use patterns of hydrogen bonds, sometimes augmented with local motif superpositions, to determine which residues are in a helix, strand, etc. These methods use cut-offs to determine if a hydrogen bond exists or not; with a cut-off of 3.50 Å, for example, a distance of 3.49 Å between the backbone N and O leads to the assignment “hydrogen bond”, while a distance of 3.51 Å does not. Obviously, this effect is strongest near the ends of secondary structure elements. It therefore makes great sense to either not care about the precise location of these ends [29], or to determine the ends of secondary structure elements by visual inspection of three-dimensional structures.

The applied crystallographic methods, from crystallization (and resulting crystal packing) to model building and refinement, influence fine details in the structure such as lengths of hydrogen bonds near the ends of helices, and thus also the secondary structure assignment by DSSP. To evaluate the magnitude of this problem, we could compare DSSP assignments for structures solved at low resolution with the same structure later solved at higher resolution, or we could compare the DSSP assignments for close homologs. It would probably be best to have the structures of a large number of proteins solved independently by several crystallography groups and compare the outcomes, but this still has the problem that a protein crystallized from a saturated ammonium sulfate solution is in a very different physicochemical environment than a protein solved from a polyethylene glycol solution. When a protein crystallizes as a dimer in the asymmetric unit, however, the two monomers in this dimer normally are covalently identical and both are certainly present in the same physicochemical environment. The only difference between the two monomers is that their crystal packing environments are different. Comparing the DSSP assignments of monomers in a dimer are thus a good way to establish to what extent DSSP assignments are the result of crystal packing artefacts and refinement strategies, and thus provide a good estimate of the very upper limit of the accuracy of secondary structure determinations, and thus of PSSP.

**Figure 3:**
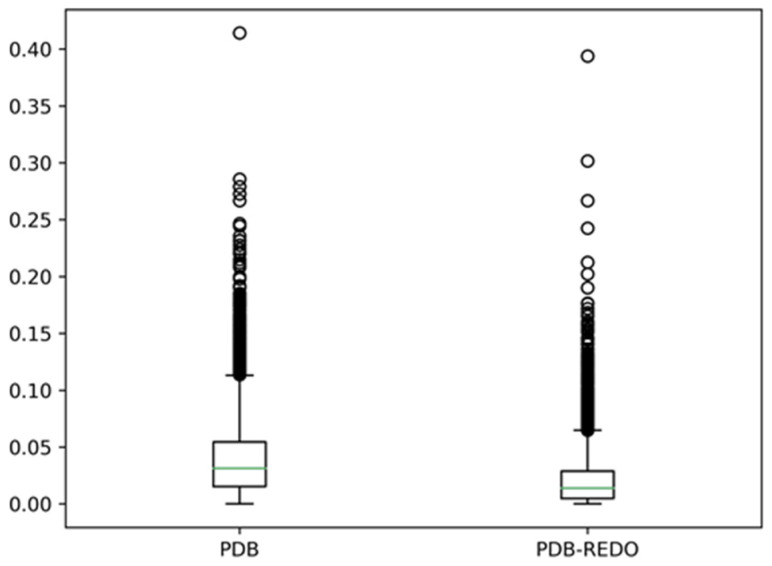
Box and whisker plots for the comparison of DSSP assignment of the A and B chains in covalently identical dimers. (Left) About 7000 dimers found in PDB files; (Right) The corresponding PDB-REDO files. The secondary structure assignments by DSSP are reduced to three states (helix, strand, and rest). Assignments are called different if the assignment character for the residues at equivalent positions in the two chains in the dimer are different.

We used WHAT IF [31] to find in the PDB all files that have two covalently identical monomers in the asymmetric unit, and asked how different those two monomers were in terms of secondary structure as assigned by DSSP. As crystallographers often force the two monomers to be identical, we did not include structures in which the two monomers could be superposed exactly. Figure 3 shows the results of comparing the eight-state assignments by DSSP for about 7000 PDB files.

Figure 3 shows that there are, on average, 3% different DSSP assignments between monomers in a dimer, and DSSP assignment differences of more than ten percent are observed for many dimers. In the PDB-REDO files, these differences are about two times smaller. Clearly, re-refinement has a big influence on the DSSP assignments, but that is beyond the scope of this article [32]. Here, it suffices to conclude that there is already a 3% difference in secondary structure depending on whether one uses either the A chain or the B chain from the same dimer, and these differences are most prominent near the ends of helices. Needless to say, better results are obtained if the secondary structure assignments for the ends of helices are either left fuzzy [29] or are determined by visual inspection, as was Oleg Ptitsyn’s preferred approach.

### 3.2. How Important Is the Size of the Training Set?

When Oleg Ptitsyn started working on PSSP, neither the PDB nor graphics capable computers existed yet, and the world knew the 3D-structure of just a couple dozen proteins. Consequently, he could only learn from a very small dataset. Upon browsing through the first 500 Google Scholar hits for “protein secondary structure”, we found several hundred articles describing a PSSP method, and what the majority of these methods have in common is the use of a training set and a test set that were selected to contain only sequence unique proteins. Many of these articles mention that at some later time, with more data available, their method will work better. This latter, however, is not applicable to the work of Oleg Ptitsyn, who wanted to understand the physics of protein structures. Physics is physics; once gravity is understood, for example, we do not need a thousand more apples to fall to better understand gravity.

**Figure 4:**
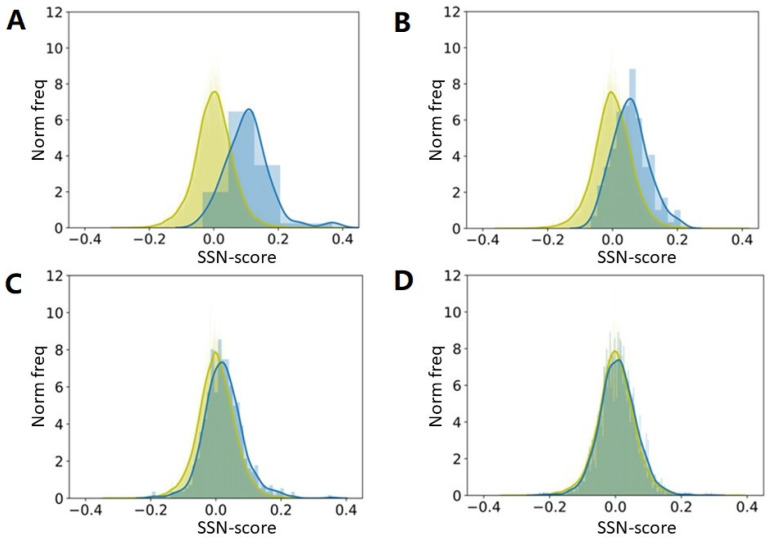
Secondary structure normality (SSN) scores for all 22,405 proteins when preference parameters are based on a small, randomly selected subset of all proteins. From (**A**–**D**) the preference parameters are determined from 50, 150, 500, and 2000 proteins, respectively (in blue). SSN scores for the proteins in the small training sets are in blue and for the complete set of 22,405 proteins in yellow. Both training and test set are shown as a normalized histogram with the width of the bins chosen to have the maximum count between 6 and 10, and similar between the two distributions. The curves are obtained from those histograms using a kernel density estimation. The abscissa is based on the SSN scores for the proteins in the training set.

We took 22,405 protein chains that are all sequence unique at the 30% level. We used preference parameters determined from a (small) subset of these 22,405 and asked how “normal” all proteins in our big dataset were. Figure 4 shows that once a few thousand non-redundant proteins are available, a further increase of the size of the training-set does not matter much for a preference parameter-based method, and we expect that this will also hold for the hundreds of PSSP methods published since 1973. This observation can be explained with the so-called bottleneck effect from evolution theory, in line with the conclusions by Kabsch and Sander [25] about the dataset used by Ptitsyn.

We were not aiming at predicting the secondary structure of proteins. We only wanted to see how well such methods could, in principle, perform. The underlying idea is that proteins with a very high SSN-score are likely to be predicted well by C&F-like PSSP methods, while proteins with a very low SSN-score will not. We repeated the experiments many times with different subsets, and got each time highly similar results. We have not Jackknifed the method, which explains (partly) why the SSN score of the proteins in the training set is higher than that of the proteins in the test set. Once the training set is large enough to no longer suffer from counting statistics problems, this effect is gone. A larger training set would have improved PSSP methods (a bit) in the 80s but by the time the PDB held many thousands of proteins, this stopped being true.

### 3.3. How Important Is the Composition of the Training Set?

**Figure 5:**
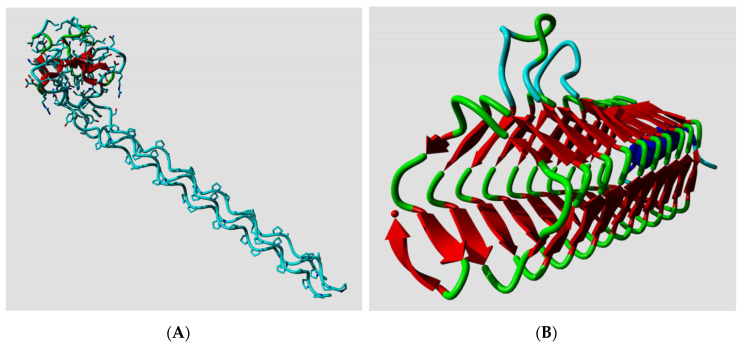
Structures with extreme secondary structures. (**A**) 1nay [36] is the protein with the highest “normality”, defined as the highest average P(aa_i_,ss_j_) + P(aa_i_,aan_i_,ss_j_,ssn_j_). This protein has a long stalk with a type of helix that DSSP recognizes as loop. This stalk is rich in prolines and P(Pro,Loop) is very high so that the normality of this protein is high. (**B**) 3du1 [37] is the protein with the lowest “normality”. The repeats in this protein are kept together by (cooperative) interactions along the tube. The strands (red) are accidentally rich in residues for which P(aa,strand) is low while the (green) hydrogen bonded turns and (blueish) loops contain residues for which this parameter is high (and thus P(aa,turn) is low).

Several hundred proteins with either the highest or the lowest SSN scores were manually analyzed. These are the proteins that are likely to perform best or worst, respectively, in PSSP projects. Figure 5 shows the protein with the highest and the protein with the lowest SSN score in the dataset.

**Figure 6:**
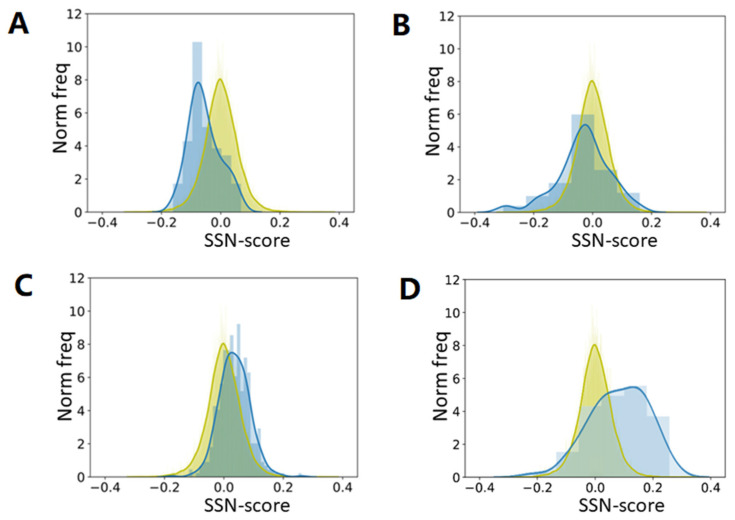
Normality scores for four classes of proteins determined using SSN scores obtained from a large, representative dataset. The green histogram is for all 22,405 proteins in the dataset, the blue histogram is for the subset. The axes are similar as in Figure 4. (**A**) The blue histogram is for 79 files with “hemoglobin” in the text. (**B**) 65 proteins with “finger” in the text (mostly zinc-finger or ring-finger). (**C**) 387 proteins with “thermophiles” in the text. (**D**) 66 proteins with “de novo” in the text.

Among the proteins with the lowest normality score, we found a large number of ubiquitins, VEGF-A proteins, and haemoglobins. Visual inspection revealed that these extremely low-scoring proteins contain many, or sometimes exclusively d-amino acids, but we could not find a trivial explanation for the large number of haemoglobins with low normality. Figure 6 shows the histograms of all 22,405 SSN scores with as insert a histogram for the SSN scores of 79 files in the dataset with “hemoglobin” either in the text of the PDBFINDER [34] entry, or in the PDB website’s “Structure Title” or “Classification” record.

Haemoglobins are among the oldest proteins for which the 3D structure was determined. Consequently, Oleg Ptitsyn and his group studied these proteins longest [1], and therefore might have understood them better and predicted their secondary structures better. These same haemoglobins, however, are likely to be predicted worse by C&F-like PSSP methods. Figure 6 indeed shows that haemoglobins tend to have a worse SSN score than all other proteins; this led us to ask if there are many groups of proteins that have aberrant SSN scores. We first looked at GPCRs as their core structure has seven helices and essentially no strands, just like the haemoglobins. GPCRs, however, get very normal SSN scores (despite being membrane proteins), as do ubiquitins, antibodies, and a whole series of protein classes that have one representative among either the lowest or the highest 250 SSN scores. The only other aberrantly scoring class we found was based on the word “finger” (zinc-fingers and ring-fingers are recognizable local folding motifs that often are mentioned in the name or the annotation of a protein).

On the other hand, if we look at all de novo proteins, we see that they have very high SSN scores, something that is not surprising, as secondary structure preference parameters undoubtedly are used in designing the sequences of these proteins. Figure 6 also shows that thermostable proteins tend to have high SSN scores (in line with old work about thermostabilizing mutations (e.g., [38])).

So, when it comes to SSN scores, there seem to be different types of protein classes, those that behave like the average, and those that do not.

**Figure 7:**
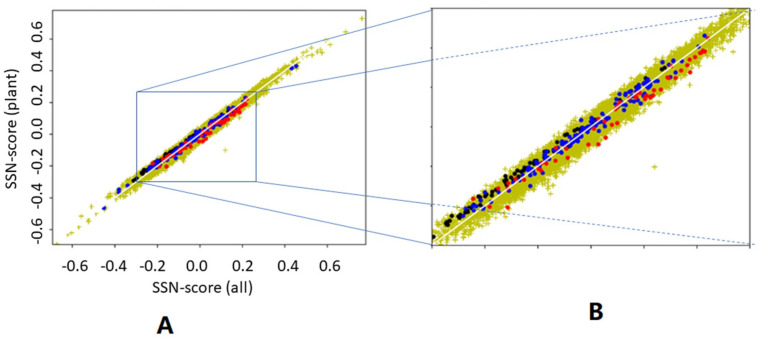
SSN scores based on plant-based versus general preference parameters. SSN scores for all 22,405 proteins were determined twice. For the abscissa, the SSN scores were determined using preference parameters extracted from all proteins, while for the ordinate, the SSN scores were determined using preference parameters obtained from 156 plant proteins. So, dots above the (white) Plant-SSN=All-SSN diagonal line represent proteins that have a higher SSN score when the preference parameters are determined from plant proteins only, while dots below the line get a higher SSN score when general preference parameters are used. Yellow: all proteins; Blue: plant proteins; Black: Haemoglobins; Red: antibodies. (**A**) the whole plot including all 22,405 proteins; (**B**) blown-up part of the left-hand side plot showing most red, blue, and black dots to better see the systematic nature of the asymmetries of these colored dots.

We asked what would happen if we determine the preference parameters from plant proteins only, and then determine the scores for all proteins. We asked this question because plant proteins are known to be different from animal proteins in many ways [39]. Figure 7 suggests that it does not matter much whether preference parameters are determined from 22,405 proteins or from just 156 plant proteins. It is surprising, however, that preference parameters determined from plant proteins give higher SSN scores for haemoglobins than preference parameters determined from all 22,405 proteins, while the opposite is found for antibodies. Both haemoglobins and antibodies are non-plant proteins that are typically found in animals only.

**Figure 8:**
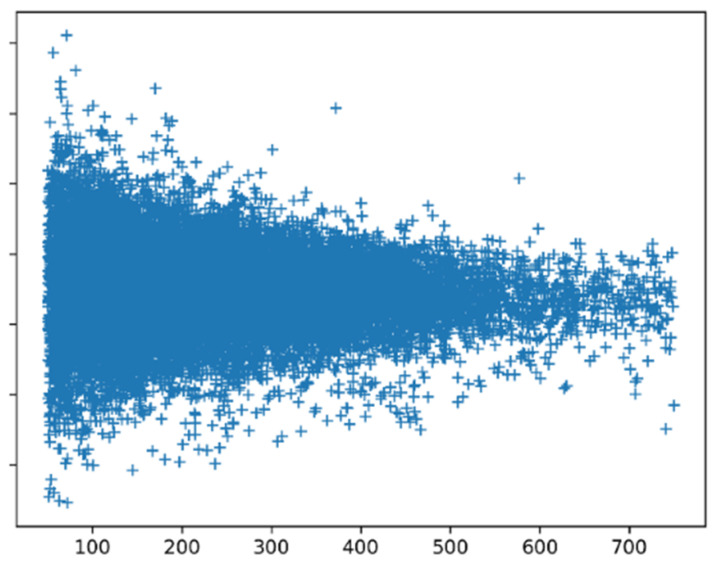
SSN scores as function of protein length.

Figure 8 shows the SSN scores for all proteins calculated with preference parameters based on all proteins. Small proteins clearly have more extreme scores than large proteins. Manual inspection of the scores of all proteins of 50–60 amino acid length revealed that among the low scoring proteins, we find many peptides that are fixated in a complex (like inhibitors bound to the enzyme they should inhibit), while among the high scoring proteins, we find mainly monomers and homomultimers. The peptides that are bound in a complex are likely to have an induced-fit structure that might not necessarily need to follow the preference parameters.

Long proteins tend to have homogeneous SSN scores, while shorter proteins are more extreme. Short proteins can be more influenced by the environment than long ones, and indeed, short proteins, when bound to something, tend to have poor SSN scores.

One of the referees suggested that we analyze natively unfolded proteins. We used a set of 225 intrinsically disordered proteins and asked the question what the SSN score would be if we predicted all amino acids as helix, as strand, or as turn.

**Figure 9:**
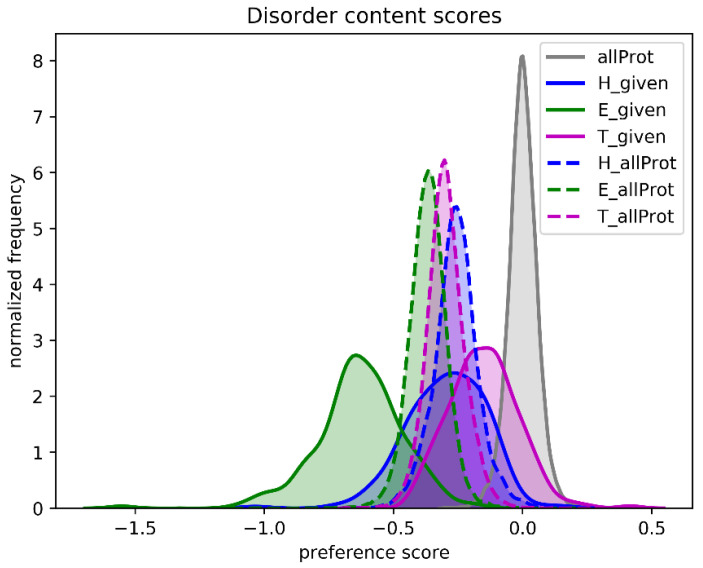
SSN scores for intrinsically disordered protein regions assuming one secondary structure. The grey curve represents the SSN scores for the full set of 22,405 proteins. The SSN scores of these 22,405 proteins were determined assuming each protein was either one long Helix (dashed blue), Strand (dashed green), or Turn (dashed magenta). Similarly, solid lines (in the same colors) show the SSN scores determined for 225 natively unfolded protein regions assuming one single secondary structure for that whole region.

Figure 9 shows that, compared to the native secondary structure, the SSN scores of all proteins decrease when one type of secondary structure is assumed. The assumption that the whole protein is one long helix gives the highest scores, while the assumption that the whole protein is one long loop gives the worst score. When the same is done for 225 intrinsically disordered proteins, the SSN scores are worst for the Strand assumption, while the scores for the Loop assumption are close to the SSN scores of folded proteins. This latter observation is not overly surprising as unfolded protein regions essentially are unstructured loops. C&F-like PSSP methods are thus expected the predict that natively unfolded proteins consist mainly of loops, with the occasional helix, and barely no strands.

## 4. Discussion

When we apply C&F-style preference parameters to determine the secondary structure normality of all 22,405 proteins in the dataset and look at the proteins with the lowest secondary structure normality, we observe several classes that, on average, are either very “normal” or very “abnormal”. “Abnormal” proteins would be predicted poorly by a C&F-like PSSP tool. Indeed, the sequence of 3DU1 (see Figure 5) was predicted by the most recent C&F prediction server added to the internet (CFSSP9) to contain much more helix than strand and loop, while Figure 5 shows that it consists of strand, loop, and turn only. Among the 500 most abnormal proteins, we find a large number of haemoglobins. When we recalculate the parameters using only 79 haemoglobins then, obviously, the haemoglobins all are considered normal, but amazingly, the large dataset of 22,405 proteins does not score very abnormal. It is clear that PSSPs based on C&F-like parameters will only work well for middle-of-the-road proteins, and this is probably true for most PSSP projects, even when based on small or medium size neural networks.

In Ptitsyn’s group, people looked at protein structures and tried to understand the principles that governed their folding and stability. Just this—“looking” at proteins using deep learning methods based on large neural networks and big data—is that what modern PSSPs try to do! Our results suggest that PSSP projects might benefit from a (fully unsupervised) protein-class prediction, followed by a prediction with a PSSP method specific for that class. These classes will likely neither be the typical all-α, all-β, α/β, α+β and further subclasses used by most protein classification schemes, nor will they always be classes of proteins with a similar function. Classes might be function-related, related to cellular location, to species, to origin, etcetera. We expect that if all kinds of technical problems, such as overtraining, local minima in neural space, or computational times required, can be overcome, then modern deep-learning based methods (with a very, very big neural network) can automatically detect these classes, and it is remarkable that (albeit without knowing it) this is rather much what Oleg Ptitsyn and his group did when they started the PSSP research field in the early 70s.
